# Histone methylation: at the crossroad between circadian rhythms in transcription and metabolism

**DOI:** 10.3389/fgene.2024.1343030

**Published:** 2024-05-16

**Authors:** Mirna González-Suárez, Lorena Aguilar-Arnal

**Affiliations:** Departamento de Biología Celular y Fisiología, Instituto de Investigaciones Biomédicas, Universidad Nacional Autónoma de México, Mexico City, Mexico

**Keywords:** histone methylation, circadian rhythms, metabolism, transcription, epigenetics, chromatin

## Abstract

Circadian rhythms, essential 24-hour cycles guiding biological functions, synchronize organisms with daily environmental changes. These rhythms, which are evolutionarily conserved, govern key processes like feeding, sleep, metabolism, body temperature, and endocrine secretion. The central clock, located in the suprachiasmatic nucleus (SCN), orchestrates a hierarchical network, synchronizing subsidiary peripheral clocks. At the cellular level, circadian expression involves transcription factors and epigenetic remodelers, with environmental signals contributing flexibility. Circadian disruption links to diverse diseases, emphasizing the urgency to comprehend the underlying mechanisms. This review explores the communication between the environment and chromatin, focusing on histone post-translational modifications. Special attention is given to the significance of histone methylation in circadian rhythms and metabolic control, highlighting its potential role as a crucial link between metabolism and circadian rhythms. Understanding these molecular intricacies holds promise for preventing and treating complex diseases associated with circadian disruption.

## Introduction

Circadian rhythms (derived from Latin “circa diem,” meaning “around a day”) are 24-h cycles that allow organisms to match their biological functions, such as behavior and physiology, to daily environmental fluctuations. For example, in mammals, feeding/fasting, sleep/wake cycles, metabolism, body temperature, and endocrine secretion are under circadian control (Bass and Lazar, 2016). This mechanism evolved in all light-sensitive organisms and provides an intrinsic and anticipatory mechanism to enable temporary responses to external cues, otherwise known as zeitgebers (a German word meaning “time-giver”) (Vaze and Sharma, 2013). The daily light–dark cycle is the best-known zeitgeber, but food intake can also entrain the circadian clock (Pendergast and Yamazaki, 2018).

Circadian rhythms can be analyzed as an integrated system that is controlled by a complex and hierarchically structured network comprised of a central clock and peripheral clocks in different tissues (Albrecht, 2012). The central clock is located in the suprachiasmatic nucleus (SCN), which consists of a set of 10,000–20,000 neurons. These neurons integrate light signals and adjust information about time to its own circadian oscillation. In return, the SCN synchronizes subsidiary peripheral clocks using an intricate network of neuronal and humoral signals that will onset certain behaviors and physiological outputs (Hastings et al., 2018). At the cellular level, the core clock molecular machinery consists of transcription factors and epigenetic remodelers that drive the circadian expression of multiple transcripts in the genome (Aguilar-Arnal and Sassone-Corsi, 2015). However, environmental signals also give feedback to the circadian clock, providing the necessary flexibility and plasticity to adjust to physiological needs (Reinke and Asher, 2019). Hence, circadian disruption leads to a wide range of diseases, including obesity, metabolic syndrome, type 2 diabetes, cardiovascular diseases, and even cancer (Kelly et al., 2018; Sulli et al., 2019). Thus, understanding the mechanisms behind circadian disruption becomes imperative to prevent and treat many complex diseases (Escalante-Covarrubias and Aguilar-Arnal, 2018).

One way of communication between the environment and chromatin is through histone post-translational modification and chromatin remodeling. In this review, we will focus on the importance of histone methylation in circadian rhythms and control of metabolism, giving special emphasis on how these modifications could serve as another link between metabolism and circadian rhythms.

### The mammalian molecular clock

**FIGURE 1:**
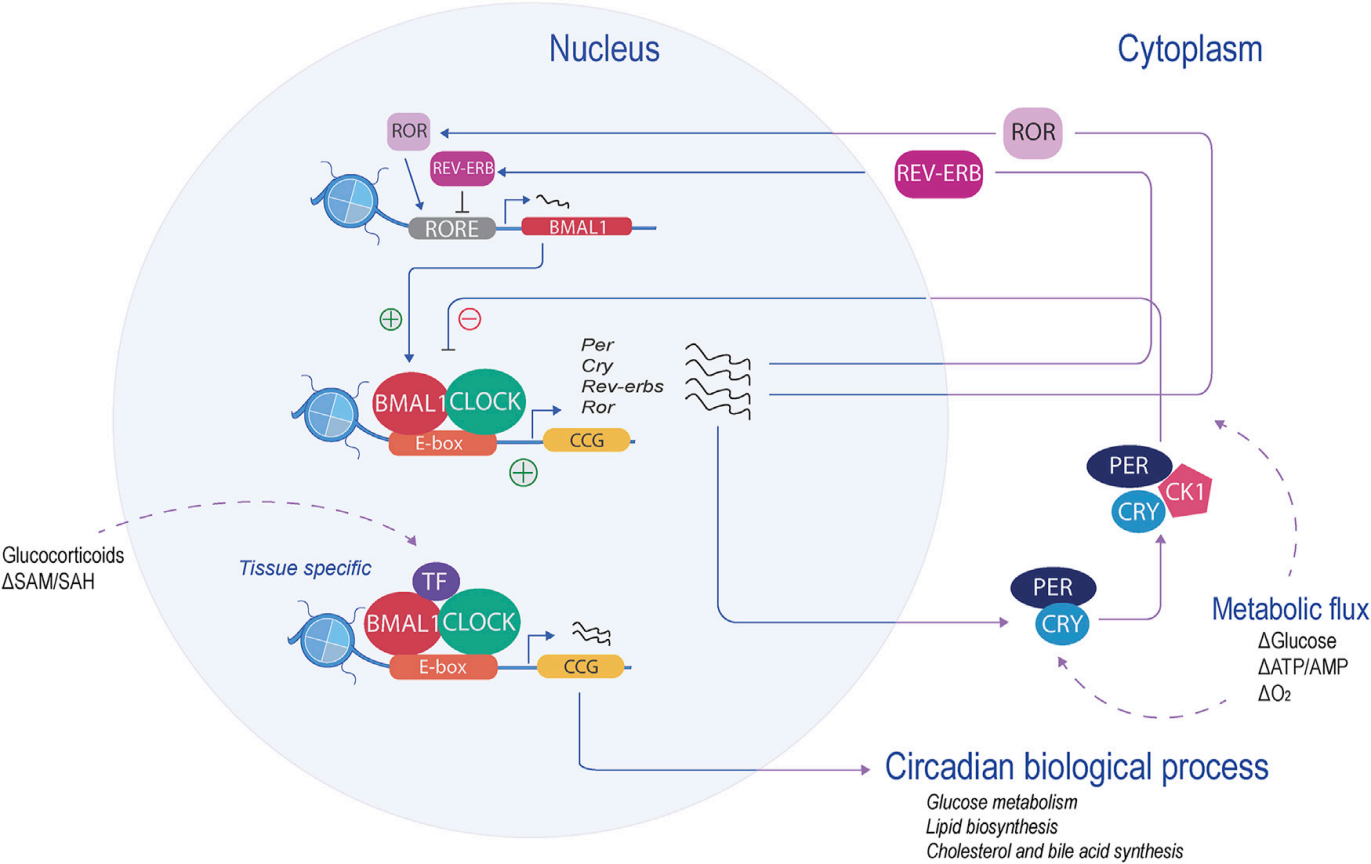
Mammalian molecular clock. At the core of the molecular clock, CLOCK and BMAL1 form a heterodimer and bind to the E-boxes (orange), activating the transcription of the CCGs (yellow). The negative regulators (period and cryptochrome) are transcribed and translated. Their product represses their own transcription. Another feedback loop, the RORE loop, regulates BMAL1 levels (red). These feedback loops, in coordination with specific transcription factors, regulate the transcription of tissue-specific CCGs and, therefore, the circadian biological process.

The mammalian molecular clock is present in almost all differentiated cells, and it coordinates rhythmicity through an autoregulatory feedback loop of transcription and translation (TTFL). This molecular mechanism is similar at the cellular level, although it exhibits distinctive physiological functions depending on the tissue. The core components of the molecular clock are transcription factors that coordinate and drive specific programs of gene expression within 24 h (Figure 1). At the core of this molecular clock, the activation is driven by basic helix-loop-helix (b-HLH)-PAS (Per-Arnt-Sim) proteins: circadian locomotor output cycles kaput (CLOCK), neuronal PAS domain protein 2 (NPAS2), and brain and muscle ARNT-like 1 (BMAL1 or ARNTL1). These proteins positively regulate the expression of many clock-controlled genes (CCGs) by pioneer binding to their E-box cis-elements and priming them for transcriptional activation (Debruyne et al., 2007; Menet et al., 2014; Trott and Menet, 2018). Among the CCGs, the negative regulators of the core clock are transcribed: period (*PER1*, *PER2*, and *PER3*) and cryptochrome (*CRY1* and *CRY2*) (Kume et al., 1999; Albrecht et al., 2001). PER and CRY proteins accumulate in the cytoplasm, reaching a stoichiometric threshold, and dimerize with casein kinase 1 delta (CK1δ), forming a repressive complex that will return to the nucleus, inhibiting BMAL1-CLOCK transcriptional activity and, therefore, its own transcription (Etchegaray et al., 2010; Lee et al., 2011). The decrease of PER and CRY will release the repression of BMAL1-CLOCK, enabling its transcription again, thus begging for a new cycle. Two other feedback loops are integrated into the core clock mechanism, providing robustness to the oscillatory mechanism. One of them is the retinoic acid-related orphan receptor-binding elements (ROREs), and the second loop is D-box elements.

The RORE loop acts as a principal regulator of *BMAL1.* This limb is coordinated by retinoic acid receptor-related orphan receptors (RORα, RORβ, and RORγ), acting as transcription activators, along with the repressors, nuclear receptor reverse erythroblastosis virus (REV-ERBα and REV-ERBβ). ROR and REV-ERB recognize and compete with the RORE sites, thereby enabling a feedback loop that regulates BMAL1 levels (Preitner et al., 2002; Sato et al., 2004). This binding mechanism is tissue-specific, allowing the circadian rhythmicity of transcripts to be tailored to the specific needs of that tissue (Fang and Lazar, 2015). The D-box feedback loop is coordinated by activators such as D-box binding protein (DBP), thyrotroph embryonic factor (TEF), hepatic leukemia factor (HFL), and the repressor, nuclear factor interleukin-3-regulated (NFIL-3, also known as E4BP4) (Gachon et al., 2006). These factors recognize, compete, and bind to the D-box motif generating circadian expression of their target, one of them being RORγ (Mitsui et al., 2001; Yu et al., 2013). Interestingly, the expression of *Dbp* is driven by the E-boxes in the opposite phase of NFIL-3, which is driven by the RORE loop (Ripperger and Schibler, 2006).

These three feedback loops interconnect with other proteins and regulate each other, creating complex clockwork, which in turn can, in combination or individually, impose rhythmicity on many genes at several amplitudes or phases in a tissue-specific manner. One example of tissue-specific oscillation is the peripheral feedback loop of the hepatocyte nuclear factor 4 alpha (HNF4α) in liver mice. HNF4α interacts with the core clock and represses the activity of BMAL1-CLOCK at specific metabolic genes (Qu et al., 2018).

### Circadian rhythms and metabolism

Metabolism is controlled by the internal circadian rhythm and, conversely, provides an important input to the circadian clock. The first evidence was from genetic studies, where a deletion or mutation of clock genes disturbed metabolic pathways and caused metabolic disorders (Lamia et al., 2008; Le Martelot et al., 2009; Cho et al., 2012; Barclay et al., 2013). For example, Turek et al. (2005) demonstrated that a mutation in the *Clock* gene in mice leads to hyperphagia, obesity, and metabolic complications such as hyperlipidemia and hyperglycemia, among others. Another example is the triple *Per* mutant mice (*Per1/Per2/Per3*), which develop obesity when fed a high-fat diet (HFD) (Dallmann and Weaver, 2010). On the other hand, the use of omics technologies has made possible the discovery of multiple circadian patterns of transcripts, proteins, and metabolites that drive cellular rhythmicity. Transcriptomic analysis in different mouse tissues demonstrated that a large proportion of the whole transcriptome undergoes circadian oscillation, particularly in the liver, a well-known tissue with multiple metabolic functions (Zhang et al., 2014a). Further studies revealed that the central nodes of the core metabolic pathways of the liver, as well as proteins, metabolites, and lipids, are under circadian control (Panda et al., 2002; Rey et al., 2011; Adamovich et al., 2014; Mauvoisin et al., 2014; Krishnaiah et al., 2017).

One principal way of circadian control of metabolism is through transcription either by direct action, controlling metabolic genes, or indirectly, by regulating nuclear receptors, thus regulating the oscillation of their targets. These transcripts include gene-controlling processes as widespread as glucose metabolism and transport (*G6Pase*, *Pepck*, *Glut2*, and *C/ebpα*), lipid biosynthesis (*Pparγ*, *Pparα*, *Lxr-α*, *PGC-1α*, *Apoc3*, *Fas*, *Chrebpα*, and *Fgf21*), and cholesterol and bile acid synthesis (*Srebf1*, *Cpt-1*, *Hmgcr*, *Cyp7a1*, and *Insig2*) (Raspé et al., 2002; Oishi et al., 2008; Le Martelot et al., 2009; Grimaldi et al., 2010; Zhang et al., 2010; Cho et al., 2012; Kawasaki et al., 2013; Abdul-Wahed et al., 2017). Consequently, chronic disruption of the circadian rhythm may lead to the development of metabolic disorders, including obesity, metabolic syndrome, and type 2 diabetes (Bedrosian et al., 2016; Mason et al., 2020).

Hence, the clock plays a key role in metabolic homeostasis; however, numerous metabolic pathways have been shown to have reciprocal feedback mechanisms to reprogram the clock. The molecular clock can sense changes in energy state and adjust its own rhythm and functions, allowing fine-tuning and temporal regulation (Feng et al., 2012). This process is achieved through proteins that can sense changes in metabolites and regulate their own activity. Examples of proteins that may provide such responses are the sirtuin family of deacetylases (SIRT1 and SIRT6), which are sensitive to NAD+/NADH levels (Liu et al., 2007; Nakahata et al., 2009; Ramsey et al., 2009; Masri et al., 2014), as well as the protein kinase AMPK, which is sensitive to the intracellular ratio of AMP/ADP (Um et al., 2007; Vieira et al., 2008; Jordan and Lamia, 2013). Other metabolites, such as SAM/SAH or 2-oxoglutarate, may also play a role in modulating different protein activities, but further investigation is required to establish their relationship with circadian control.

It is important to state that many of these metabolites are subjected to food availability (feed/fasting cycle) or diet composition. Thus, one dominant zeitgeber for metabolically active tissues, such as liver and adipose tissue, is feeding patterns. It is now well-known that the hepatic clock arises from an interaction between feeding cues and the circadian clock itself (Hatori et al., 2012). Therefore, changes in these cues can affect the circadian clock. For example, HFD lengthens the circadian period and abolishes metabolites and transcriptional rhythm in mice, but, on the contrary, *de novo* transcripts and metabolites emerge, rewiring the circadian clock (Kohsaka et al., 2007; Eckel-Mahan et al., 2013). Yet, if this HFD or other obesogenic diets are time-restricted, the consequences are far less severe, with protection against hyperinsulinemia, obesity, hepatic steatosis, and changes in the oscillation of circadian clocks (Hatori et al., 2012; Chaix et al., 2014). Much of this effect is through the regulation of transcription factor activity such as PPARγ, SREBP-1, and PPARα (Eckel-Mahan et al., 2013; Guan et al., 2018), but there are also indirect ways in which metabolism can influence circadian transcription, especially at the level of the chromatin fiber and *vice versa*.

### Circadian rhythms and chromatin remodeling

DNA in the eukaryotic cell is stored in a structure that allows the cell to perform specialized functions and respond to changing environments. This highly organized structure is called chromatin and is predominantly arranged in repeating units of nucleosomes. The nucleosome is the fundamental unit of chromatin and consists of ∼147 pb of DNA wrapped around a histone octamer containing two copies of H2A, H2B, H3, and H4. Each nucleosome is linked to an adjacent nucleosome through a fragment of linker DNA in association with a linker histone (H1 or H5) (McGinty and Tan, 2014). The storage and accessibility of the genome are regulated at this level through post-translational modifications (PTMs) of the histone tails that alter chromatin structure and function. These modifications are generated by epigenetic/chromatin regulators that can “write,” “erase,” and “read” the histone tails. Distinct PTM generates a “histone code,” creating distinct chromatin states that can be translated into biological functions (Jenuwein and Allis, 2001).

**FIGURE 2:**
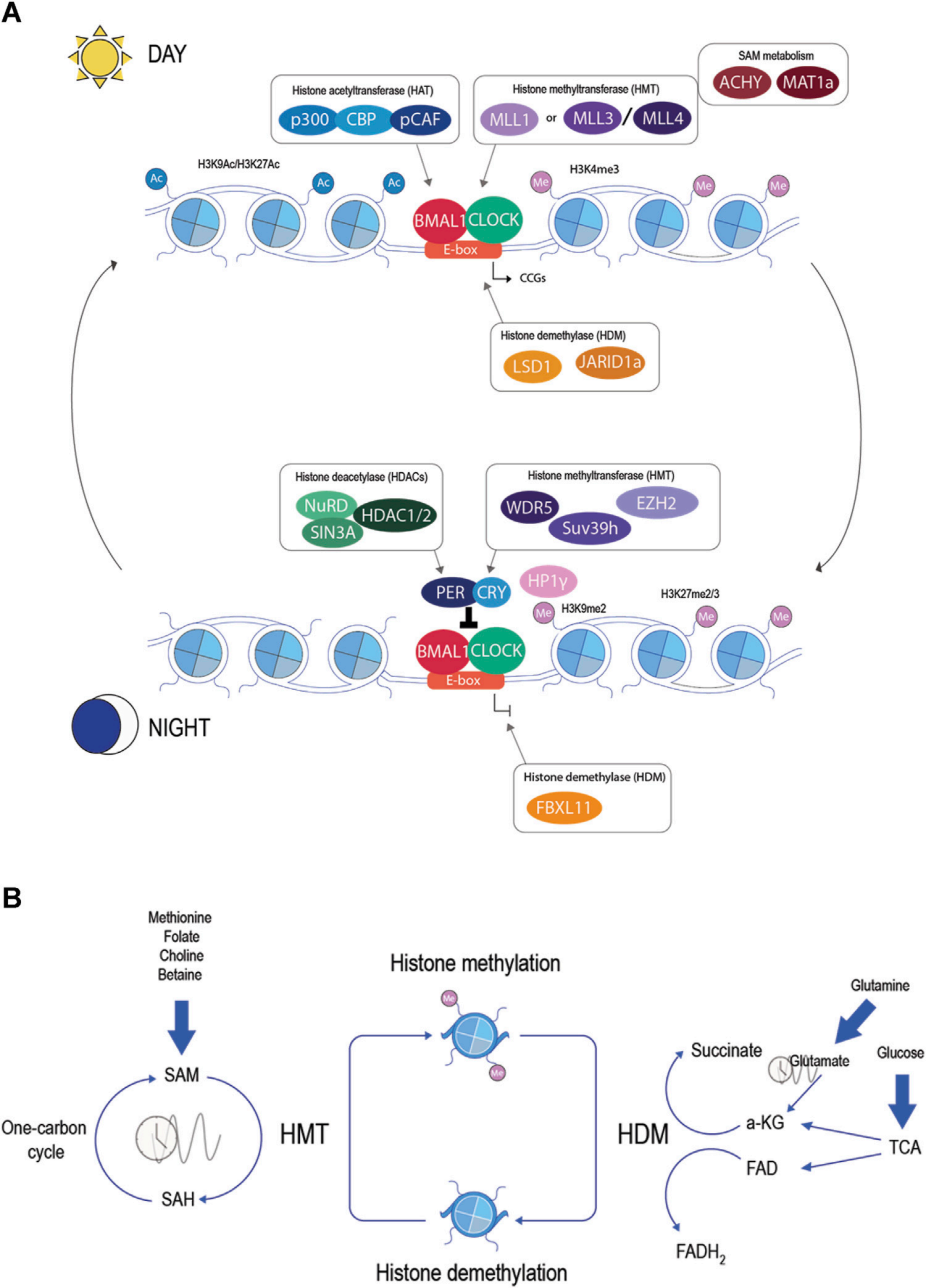
**(A)** Epigenetic regulators in the molecular clock. During the active phase (day), the binding of CLOCK and BMAL1 is associated with the recruitment of chromatin factors: histone acetyltransferase (blue), histone methyltransferase (violet), histone demethylase (orange), and metabolic enzymes (brown). In the repressive phase (night), other chromatin factors will be associated with the molecular clock: histone deacetylase (green), histone methyltransferase (violet), and histone demethylase (orange). These chromatin factors will raise sequential cycles of active-repressive marks in the histones and generate rhythmic transcription of CCGs. **(B)** Histone methylation and metabolites. Histone methylation is catalyzed by histone methyltransferase and erased by histone demethylase. HMT uses SAM as a donor of the methyl group, creating SAH. SAM is produced in the one-carbon cycle and can be influenced by nutrients such as methionine, folate, choline, or betaine. The ratio of SAM/SAH shows circadian rhythms. HDM uses α-ketoglutarate or FAD as a cofactor. α-KG is an intermediate of the tricarboxylic acid cycle (TCA) or can be produced by glutamate, a circadian metabolite. FAD is a product of the TCA and is directly influenced by the redox state.

In the past two decades, research has been made to decipher the basic epigenetic regulation to understand gene expression in different biological systems, including circadian rhythm. It is now accepted that circadian machinery needs the assistance of many chromatin factors to regulate circadian gene expression; thus, transcriptional cycles occur at the level of chromatin fiber through tight cooperation and reciprocal regulation between clock proteins and transcriptional regulators (Pacheco-Bernal et al., 2019) (Figure 2A). Indeed, the core machinery itself directs the activity of multiple chromatin factors to regulate histone post-translational modifications implicated in transcriptional control. As an example, CLOCK itself is a histone acetyltransferase (HAT), targeting lysine 9 and 14 (H3K9/H3K14), and acts in concert with p300, CREB-binding protein (CBP), and CBP-associated factor (pCAF) (Takahata et al., 2000; Doi et al., 2006; Lee et al., 2010). The counteracting part are the histone deacetylases (HDACs) which act during the repressive phase by deacetylating histones and repressing transcription. PER-CRY associates with Mi-2/nucleosome remodeling and deacetylase (NuRD), followed by the recruitment of SIN3A and HDAC1/2 (Naruse et al., 2004; Duong et al., 2011; Kim et al., 2014). Chromatin immunoprecipitation followed by deep sequencing (ChIP-seq) has shown rhythmic histone acetylation at H3 lysine 9 and lysine 14 (H3K9/H3K14) and also histone methylation at H3 lysine 4 and lysine 27 (H3K4/H3K27), rising sequential cycles of active-repressive marks (Koike et al., 2012; Vollmers et al., 2012). These studies indicate an intricate relationship between the core clock genes and chromatin remodelers.

### Histone methylation in circadian rhythms

Histone methylation is catalyzed by histone methyltransferases (HMTs) and is commonly methylated in the ε-amino group of basic lysine residues. The most extensively studied histone methylation sites include histone H3 lysine 4 (H3K4), H3K9, H3K27, H3K36, H3K79, and H4K20, in which each lysine can support three methylation states (-mono, -di, and -trimethylation) (Greer and Shi, 2012). The chromatin state varies depending on the methylated residue, ranging from active transcription sites to constitutive heterochromatin (Hyun et al., 2017). This diverse functionality is facilitated by unique reader proteins that require specific methylation states to increase their affinity toward DNA, enabling different effects on the chromatin. In circadian rhythms, different histone lysine methylation and HMT have been implicated; for example, the lysine methylation of histone H3 at Lys4 (H3K4me3), a mark of active transcription, is present with H3K9ac rhythmical in CGGs (Etchegaray et al., 2003; Ripperger and Schibler, 2006). This histone mark is catalyzed by HMT mixed lineage leukemia 1 (MLL1), which is a subunit of a large chromatin modification complex that includes other critical regulators such as WD repeat-containing protein 5 WDR5 (Song and Kingston, 2008). MLL1 interacts with BMAL1-CLOCK. This dimer is responsible for its recruitment to the chromatin and activation by SIRT1, which contributes to the circadian oscillation of H3K4me3 at CCG promoters (Katada and Sassone-Corsi, 2010; Aguilar-Arnal et al., 2015). WDR5 has also been shown to interact with PER1 and PER2, and its loss abolishes H3K4 methylation at the promoter of Rev-erbα but not other clock genes (Brown et al., 2005). Other members of the MLL family have been shown to play a role in circadian transcription. MLL3 and MLL4, as well as H3K4 HMT, direct a percentage of rhythmic transcription in liver mouse tissue (Valekunja et al., 2013; Kim et al., 2015). Together, this result suggests that different complex interactions of chromatin factors regulate the chromatin in a temporal and spatial context in association with different collaborative partners (Kim and Lazar, 2020). Another lysine methylation that plays a role in circadian regulation is H3K9me2/3, which promotes a local repressive chromatin state. This mark assists the TTFL in aiding PER repressive function, following deacetylation of H3K9 by HDAC1. PER recruits the HMT SUV39H1/2 in complex with HP1γ to circadian targets such as the *Per1* promoter and methylates H3K9 (Duong and Weitz, 2014).

Additionally, H3K27me3, a heterochromatin mark, has also been implicated in the clock function. HMT EZH2, a polycomb group enzyme, interacts with the BMAL1–CLOCK complex in the liver and methylates H3K27 at *Per1* and *Per2* promoters. EZH2 enhances CRY transcription repression, and the loss of EZH2 disrupts *Per2* and *Bmal1* rhythms (Etchegaray et al., 2006). Even though BMAL1 and CLOCK are known as transcriptional activators, it seems that EZH2 can interact with BMAL1 and act as a repressor for specific loci in Ly6Chi monocytes (Nguyen et al., 2013). The implication of H3K27me3 and EZH2 in the TTFL is not fully understood, and the mechanism in the circadian rhythm seems to be cell-type-specific. Further investigation needs to be conducted to determine its implications for circadian regulation.

Furthermore, the elongation marks H3K36me3 and H3K79me2 have been described in the gene body of CCG with delayed phase and low amplitude, reflecting the elongation process of the circadian transcriptome (Koike et al., 2012; Le Martelot et al., 2012). HMT SET domain containing 2 (SETD2) is the sole methyltransferase to catalyze the trimethylated H3K36, and its expression is altered in the forced desynchrony of the sleep–wake cycle in the human blood transcriptome (Archer et al., 2014; Huang and Zhu, 2018). In *Neurospora*, H3K36me3 HMT Set2 is required to establish the permissive chromatin state by recruiting histone deacetylase to the *frequency* (*frq*) locus and maintaining the negative circadian feedback loop (Sun et al., 2016). Even though H3K36me3 has been described as a result of the process of elongation, the mark may act as a safeguard for the circadian transcriptome.

Histone methylation was considered a stable, static modification until the discovery of the first histone lysine demethylase (HDM) in 2004 (Shi et al., 2004). Following by the discovery of the key signature of demethylating enzymes, the JmjC (jumonji C) domain, the identification of multiple histone demethylases was made (Tsukada et al., 2006). The removal of histone methylation is essential for the observed oscillation in circadian chromatin remodeling. Over the years, multiple HDMs have been described as key components of the molecular clock. One of them is HDM lysine-specific demethylase 1 (LSD1), a flavin adenine dinucleotide (FAD)-dependent demethylase of H3K9/H3K4. LSD1 is phosphorylated in a circadian manner by PKCα, which is triggered by acute photic stimuli. Phosphorylated LSD1 stimulates binding and BMAL1–CLOCK transcription activity in the *Per2* and *Dbp* loci. However, the LSD1 mutant was not affected in H3K9me2 and H3K4me2; instead, H3K9ac seems to be affected (Nam et al., 2014). LSD1 plays a dual role in the activation or repression of transcription, depending on the interacting partner (Perillo et al., 2020). Interestingly, LSD1 interacts with SIRT1, histone deacetylase 3 (HDAC3), and SUV39H2, known as circadian components (Mulligan et al., 2011; Piao et al., 2015; Nanou et al., 2017). Further studies on LSD1 are needed to elucidate the mechanism to control dynamic circadian expression and interplay with acetylation status.

Likewise, Jumonji C (JmjC) and ARID domains containing histone lysine demethylase 1a (JARID1a or KDM5a) form a complex with CLOCK-BMAL1 and are recruited in the *Per2* promotor, where they inhibit HDAC1 and enhance H3K9ac and transcriptional activation. It seems that JARID1a demethylation activity is dispensable for circadian rhythms, but the overexpression of the JmjC domain represses *Per* transcription, suggesting that, *in vivo*, JARID1a may mediate the demethylation of H3K4me3 (Ditacchio et al., 2011). Further investigation needs to be addressed to establish whether this demethylation is occurs in a time-dependent manner. Another KDM that has been implicated in circadian rhythm is lysine demethylase 2A (KDM2A or FBXL11), which demethylases H3K36me2. Through shRNA-based screening, it was identified to be a clock component that acts as a repressor of BMAL1-CLOCK-mediated transcription. It seems that this effect is mediated *via* union with DNA by its CXXC domain, but its mechanism is still elusive (Reischl and Kramer, 2015). The knockdown of KMD2 in mammalian cells and *Drosophila* affects circadian rhythm and period without affecting the circadian core clock, suggesting an unknown circadian output (Zheng et al., 2018). Additionally, JmjC domain-containing protein 5 (JMJD5) has been identified as a regulator of the circadian system in plants and mammals (Jones et al., 2010; Shalaby et al., 2018). In mammals, JMJD5 is recruited to the CRY1–FBXL3 complex and facilitates CRY1 instability and further interaction with the proteasome for degradation. This function also seems to be dispensable from its catalytic activity (Saran et al., 2018). Other 9 JmjC domain proteins have been described in the regulation of circadian rhythms and sleep in *Drosophila*. The loss of every HDM affects the circadian rhythm in a specific manner. Together, these data imply that JmjC proteins modulate different aspects of circadian rhythm, but their mechanism is still unknown (Shalaby et al., 2018). Despite the discovery of multiple HDMs implicated in circadian regulation, the demethylation of histone of CCGs is still a puzzle with multiple intersections alongside other PTM and chromatin remodeling factors, such as BRAHMA or DOMINO (Kwok et al., 2015; Liu et al., 2019). Therefore, further research is necessary to unravel these complex mechanisms.

In addition, another level of regulation of circadian rhythms is temporal and function regulation within the nucleus. Protein levels fluctuate in the nucleus and seem to correspond to their activity peaks. A proteomic study covering over 500 proteins showed diurnal regulation. Noteworthy, several of them were HMTs and HDMs. Some of them already described having a direct implication with circadian rhythms, such as MLL3, MLL4, and JARID1a. However, there are several others without direct relation but with metabolic functions like G9a, SETD8, and JMJD1C (Wang et al., 2017). Further investigation is needed to determine whether these oscillating rhythms in the nucleus also correspond to their peak activity in their loci of regulation.

### Metabolites involved in histone methylation control: time-and diet-regulated

Chromatin-modifying enzymes, such as HMT and HDM, require intermediary metabolites as substrates or cofactors to generate chromatin modifications. Thus, by creating a complex link between metabolism and the epigenome, this relationship allows the communication of environmental changes to the chromatin (Cavalli and Heard, 2019) (Figure 2B).

One shared metabolite for all HMTs is S-adenosylmethionine (SAM), a coenzyme involved in the donation of the methyl group to the ε-amino group of the lysine residue (Finkelstein and Martin, 1986; Bannister and Kouzarides, 2011). SAM is produced by a metabolic network composed of the folate and methionine cycle, also known as the one-carbon cycle. The intracellular amount of SAM depends largely on the essential amino acid methionine. Methionine adenosyl transferases (MAT1a and MAT2a) produce the conversion of methionine to SAM (Markham and Pajares, 2009). HMTs utilize SAM to methylate their substrate and, in exchange, produce S-adenosylhomocysteine (SAH). It should be noted that SAH is a potent inhibitor of most methyltransferases, acting as a competitive inhibitor (Richon et al., 2011). SAH is then converted to homocysteine (hCys) by s-adenosylhomocysteine hydrolase (ACHY). Finally, to complete the cycle, hCys can be remethylated to produce methionine by 5-methyltetrahydrofolate (5-mTHF) or betaine-homocysteine S-methyltransferase (BHMT) (Mentch and Locasale, 2016). Noteworthy, mRNA levels of components of the one-carbon cycle, such as *Bhmt*, *Mtrr*, *Mat1*, and *Mat2*, show daily rhythms in hepatocytes (Panda, 2016). In parallel, levels of methionine, SAM, and SAH show circadian periodicity in the liver and cultured cells (Xia et al., 2015; Krishnaiah et al., 2017; Zhang et al., 2018). This evidence suggests a relationship between circadian rhythm and one-carbon metabolism. In recent years, new discoveries have tightened this relationship. For example, *Per1* and *Per2* double KO mice lose the circadian rhythm of SAH and the SAM/SAH ratio (Xia et al., 2015). Furthermore, treatment with 3-deazaadenosine (DAA), an inhibitor of SAH hydrolysis, elongates the circadian period in cultured cells and mice (Fustin et al., 2013). The inhibition of ACHY with 3-deazaneplanocin A (DZnep) affects circadian rhythms in bacteria and humans. The oscillation of circadian core clock genes damped after treatment with DZnep but could be rescued with 5′-methylthioadenosine/S-adenosylhomocysteine nucleosidase (MTAN), a prokaryote enzyme that cleaves SAH to adenine and S-ribosyl homocysteine (Fustin et al., 2020). This result suggested that the accumulation of SAH and, consequently, the reduction of methylation potential by the inhibition of methyltransferase alter the circadian rhythm. This came in agreement with the discovery of ACHY as part of the complex of BMAL1–CLOCK. Greco et *al.* demonstrated that ACHY is essential for the cyclic oscillation of H3K4me3 and, consequently, circadian transcription. The interaction of AHCY and BMAl1 is more prominent in ZT8 during the circadian transcriptional activation phase, and the inhibition of ACHY decreased BMAL1 recruitment to its target (Greco et al., 2020). Intriguingly, H3K27me3 was not altered by ACHY inhibition, suggesting a fine-tuned mechanism of the regulation of HMTs that could be associated with the localized activity of ACHY, compartmentalization of metabolites in the nucleus, or different K_m_ of the HMTs.

On the other hand, HDMs use different metabolites depending on the catalytic mechanism. Lysine-specific demethylases (LSDs) utilize FAD to facilitate methyl group removal, producing FADH2. However, JmjC domain-containing demethylases use Fe(II) and α-ketoglutarate (α-KG) (Kooistra and Helin, 2012). FAD is a redox coenzyme consisting of a riboflavin (vitamin B_2_) bound to a phosphate group of ADP. Starting from riboflavin, flavin mononucleotide (FMN) is first generated by riboflavin kinase (RFK). FMN is then converted to FAD by FAD synthase (FLAD) (Giancaspero et al., 2013). Levels of FAD and its biosynthetic enzyme RFK are rhythmic in the nucleus and regulate CRY protein stability, leading to an increase in protein levels. The knockdown of *Rfk* and a riboflavin-deficient diet altered CRY levels and the expression of Clock and CCGs (Hirano et al., 2017). In addition, the redox state is under circadian rhythms, suggesting that the ratio of oxidized to reduced FAD (FAD/FADH2) is also likely to cycle (Pritchett and Reddy, 2017). α-KG or 2-oxoglutarate is a tricarboxylic acid cycle (TCA) intermediate, coming after isocitrate and before succinyl-CoA. Several TCA enzymes have been implicated in circadian rhythms, suggesting a possible circadian flux of α-KG (Reddy et al., 2006; Eckel-Mahan et al., 2012; Vollmers et al., 2012). Alternative sources of α-KG are amino acids, especially glutamine. Glutamate, also a circadian metabolite, is deaminated to a-KG via glutamate dehydrogenase (GDH) (Krishnaiah et al., 2017). The lack of GDH in the liver has been shown to modify the circadian rhythm of food intake (Karaca et al., 2018).

Thus, glucose and glutamine catabolism maintain high levels of α-KG. In naïve ESCs, the direct manipulation of the αKG/succinate ratio is sufficient to regulate multiple chromatin modifications such as methylation in H3K9, H3K27, H3K36, and H4K20 (Carey et al., 2015). Dietary supplementation of glutamine in cancer cells increases the demethylation of H3K4me3, inhibiting oncogenes, and surprisingly, the knockdown of JARID1a rescued cancer phenotypes (Ishak Gabra et al., 2020). In the same way, the SAM/SAH ratio directly influences the levels of histone methylation. This was first visualized in mouse pluripotent stem cells, where the depletion of threonine, an amino acid involved in one-carbon metabolism, decreased the SAM/SAH ratio and H3K4me3 (Shyh-Chang et al., 2013). Moreover, methionine itself can modulate the SAM/SAH ratio and, consequently, the level of histone methylation (H3K4me3). Diet modulation of methionine led to changes in metabolism and histone methylation in the liver (Mentch et al., 2015). Other metabolites, such as folate, choline, and betaine, modulate SAM levels as well (Mafra et al., 2019). This evidence suggests that HMT and HDM can be considered metabolic “sensors,” and their regulation, activity, and subsequent gene expression regulation may be linked to the metabolic milieu. It has been proved that a changing metabolic state can influence circadian rhythms by modulating the activity of the core machinery. For example, SIRT1 deacetylase activity is coupled to the hydrolysis of the metabolite NAD+ (nicotinamide adenine dinucleotide, oxidized) and its oscillation. Because SIRT1 also regulates clock function, it is considered a metabolic sensor that connects the intracellular energy state to the clock (Escalante-Covarrubias and Aguilar-Arnal, 2018). One clear example is the regulation of MLL1 activity by SIRT1; acetylation of MLL1 affects its methyltransferase activity, consequently influencing the transcription of CCGs (Aguilar-Arnal et al., 2015). The first step has been taken to uncover the direct regulation of histone methylation and circadian rhythms with the discovery of ACHY and MAT1a as part of the molecular clock. However, further investigation is needed to determine the mechanisms by which metabolic signaling affects circadian rhythm and whether HMTs/HDMs can modulate this regulation.

### Metabolic regulation by histone methylation

The dependence of HMTs and HDMs on metabolites and their activities that are sensitive to the metabolic milieu suggest feedback regulation control. Teperino et al. (2010) suggested that HMTs and HDMs must reciprocally influence metabolism through transcriptional regulation of metabolic enzymes linking metabolism with transcription, and as predicted in the last decade, several HMTs and HMD have been described as playing a central role in the control of metabolism. In particular, HDM and HMT are implicated in the control of circadian rhythm. In this section, we will discuss the role of histone methyltransferase/demethylase, previously described, as well as others with relationships to the circadian metabolic process or oscillating key regulators in metabolism.

### Histone methyltransferases in metabolism

The first HMT to be studied in metabolism was MLL3. MLL3 −/− mice are surprisingly resistant to high-fat diet-induced steatosis, have a decrease in white fat mass, and have improved glucose tolerance and insulin sensitivity. MLL3 with ASC-2 is required for the activation of LXR-target gene expression (*FAS* and *SREBP-1c*), thus acting as coactivators of LXRs (Lee et al., 2008). In addition to MLL3, MLL4 functions as a coactivator; it associates with RORα and RORγ and activates their circadian targets, in particular the genes that maintain bile acid homeostasis (Kim et al., 2015). MLL4 has also been described as a critical regulator of overnutrition and murine hepatic steatosis. MLL4 with ABL1 associates with PPARγ2 and induces its activation in response to lipid excess (Kim et al., 2016). At last, the MLL family, MLL1 and MLL2, in coordination with ERs, have been shown to regulate cholesterol uptake by the liver (Ansari et al., 2013).

In vascular tissue, H3K9-editing enzymes SUV39H1, SRC-1, and JMJD2C drive transcription of the mitochondrial adapter p66^Shc^, a modulator of oxidative stress. Dysregulation of this enzyme was seen in obese subjects in correlation with high levels of ROS (Costantino et al., 2019). In addition, SUV39H1 has been suggested to participate in 3T3-L1 adipogenesis. In the early stages of adipogenesis, it regulates *Wnt10a*, an anti-adipogenic regulator, promoting adipogenic gene expression (Jing et al., 2020). However, with G9a, it enhances activator protein 2α (AP-2α) repression of C/EBPα in preadipocytes, thus inhibiting adipogenesis (Zhang et al., 2014b). The changes in its mechanism may be tightly regulated depending on its metabolic milieu; further investigation is needed to understand this change. In line with these observations, G9a and EZH2 have also been shown to regulate adipogenesis by inhibiting *PPARγ* or *Wnt* gene expression, respectively (Wang et al., 2012; Yi et al., 2016). Furthermore, in muscle, G9a regulates myokine, musculin, and phosphorylation of FOXO/FOXO1 (Zhang et al., 2020). EZH2 also regulates lipid metabolism in adipocytes by regulating apolipoprotein E (*ApoE*). Deletion of EZH2 resulted in hypertrophy of adipocytes and changes in plasma VLDL-triglyceride levels (Yiew et al., 2019).

The following HMTs are not involved in circadian rhythms directly but have been implicated in the regulation of circadian metabolic regulators. First, SETDB2, a H3K9 HMT, is regulated by glucocorticoids and has higher expression during a fasted state. Glucocorticoid receptor (GR) in association with SETDB2 targets *Insig2a,* a negative regulator of lipogenesis. Intriguingly, H3K9me3 was reduced and H3K4me3 was elevated at promoters of GR-target genes in the mouse liver. SETDB2 may act as a scaffold for GR to its target, thereby acting as a coactivator (Roqueta-Rivera et al., 2016). This mechanism has been shown with G9a, suggesting another mechanism of regulation of HMT (Bittencourt et al., 2012). G9a or EHMT2, an H3K9me1/2 HMT, has also been associated with the regulation of bile acid metabolism and cholesterol by modulating the expression of *Cyp27a1* and *Hmgcr* (Lu et al., 2019). Interestingly, G9a interacts with NFIL3, a circadian component of the Dbp-box loop, to inhibit *Fgf21* during refeeding conditions (Tong et al., 2013). Furthermore, G9a gains circadian oscillation at the transcript level during HFD possibly participating in the rewiring of the circadian transcriptome (Eckel-Mahan et al., 2013).

In brown adipose tissue, SUV420h is upregulated during adipogenesis (Son et al., 2016). Furthermore, SUV420h inhibits *PPARγ* transcription by H4K20me3 in brown adipocytes and has been shown to regulate metabolism and body weight in response to environmental stimuli (Pedrotti et al., 2019). In addition, Nsd2, an H3K36me1/me2 HMT, plays a role in adipogenesis. Depletion of H3K36me impairs adipose tissue development and adipogenesis by upregulating H3K27me3 in C/EBPα and other PPARγ target genes (Zhuang et al., 2018). In addition, Nsd2 is downregulated in diabetes mellitus type 2 and has been shown to play a role in insulin secretion and glucose metabolism by regulating hexokinase 2 (HK2) and glucose-6-phosphate dehydrogenase (G6PD) (Wang et al., 2016; Shi et al., 2018). At last, SETD8 or KMT5A, an H4K20me1 HMT, regulates the escape of RNA pol II from promoter-proximal regions of several metabolic genes that also coincide with the targets of PPARα (*Fasn*, *Gck*, *Hmgcr*, *Acc1*, and *Fgf21*) (Nikolaou et al., 2017). Noteworthy, PPARγ and RXRα target the SETD8 promoter and upregulate its transcription during adipogenesis (Wakabayashi et al., 2009). These results indicate a possible limb in the regulation of PPAR targets by SETD8.

### Histone demethylases in metabolism

Similar to HMT, HMD has been linked to the control of metabolism in the last few years. Despite the controversy surrounding HDMs in the regulation of the circadian rhythm, their role in metabolism seems to be clearer.

One clear example of the circadian regulation of energy metabolism and response of metabolic cues was recently published by DiTacchio et al. They described that *Jarid1a* ^*LKO*^ mouse livers loss expression of circadian pattern genes implicated in amino acid catabolism and lipid uptake, and these mice are unable to transition from fasted to fed-state gene expression. It is demonstrated that JARID1a functions as a regulator of the hepatic clock but also allows responding to feeding cues through key transcription factors (Ditacchio et al., 2020). How JARID1a can sense and alter the functions of these transcription factors is not defined, but it opens a new panorama of epigenetic regulator’ mechanisms in the interaction of the hepatic circadian transcriptome. Previously, LSD1 has also been implicated with SRBEP-1, a well-known transcriptional integrator of circadian and feeding cues. LSD1 and SIRT1 activate the SREBP-1α promoter. Later, LSD1 interacts with SREBP-1 and is needed for binding and activation of SREBP1-target genes such as *FAS* (Abdulla et al., 2014). Furthermore, the activity of LSD1 is dependent on FAD availability, linking its activity to energy sensing and thus acting as a metabolic sensor. An increase in FAD potentiates LSD1 to repress energy-expenditure genes such as *PGC-1α* through H3K4 demethylation, whereas LSD1 inhibition results in the expression of energy-expenditure genes associated with mitochondrial metabolism (Hino et al., 2012). All these results suggest that LSD1 can also modulate the activity of the core machinery depending on the metabolic state and may participate with SREBP-1 and PPARα in the rewiring of the hepatic circadian transcriptome of lipid genes in diet-induced obesity.

KDM2A HMD has also been associated with the regulation of hepatic gluconeogenesis by demethylating H3K36me3 on the *C/EBPα* promoter. The knockdown of KDM2A deregulates PEPCK and glucose-6-phosphatase (G6Pase) (Pan et al., 2012). It seems that the regulation of H3K36 methylation regulates glucose metabolism. It is tempting to speculate that an axis of KDM2A and NSD2 can drive the transcriptional regulation of glucose metabolism and may act in a circadian manner. Several other KMDs, which are not directly related to circadian rhythms, have been implicated in metabolism. One of them is the other member of the LSD family. LSD2 had been implicated in the repression of key lipid metabolic genes through the demethylation of H3K4. Interestingly, during excessive lipid influx, it may protect the cells by limiting the influx and metabolism of lipids (Nagaoka et al., 2015). Another HMD with similar protection from lipotoxicity is plant homeodomain finger 2 (PHF2). PHF2 interacts with the transcription factor carbohydrate-responsive element binding protein (ChREBP) and erases H3K9 methylation of its target genes. During metabolic stress, it increases the defense against oxidative stress and modifies lipid composition (Bricambert et al., 2018). In addition, PHF2 has been shown to be activated in a glucagon-PKA-dependent manner and act as a coactivator of HNF4α in the liver of fasted mice (Baba et al., 2011), suggesting a possible role in the circadian feedback loop of HNF4α in the liver.

Another HDM activated by PKA is Jumonji domain-containing 1A (JMJD1A or KDM3A), which catalyzes H3K9 demethylation. It has been shown that JMJD1A interacts with SWI/SNF and is bound to PPARγ to regulate thermogenic genes in brown adipocytes. This mechanism is independent of the demethylase activity by creating a scaffold that enables long chromatin interaction after an environmental cue (Abe et al., 2015). In hepatic cells, JMJD1A also seems to regulate PPARγ expression but through H3K9 demethylation (Jiang et al., 2015). Along the same lines, fasting induces PKA signaling and activates HDM Jumonji D3 (JMJD3 or KDM6B). JMJD3 interacts with PPARα and SIRT1 and activates β-oxidation genes (*Cpt1*, *Pgc-1α*, and *Fgf21*) by demethylating H3K27me3 (Seok et al., 2018). Another study by the same group linked nutrient deprivation to histone modifications and transcriptional induction of hepatic autophagy in mice by the JMJD3–FGF21 axis (Byun et al., 2020). Furthermore, HDM has been described in the control of metabolism, suggesting a high potential for HDMs as metabolic sensors (Zhao et al., 2016; Kang et al., 2018; Backe et al., 2019; Viscarra et al., 2020).

## Conclusion and future remarks

Histone methylation has been previously described as an important part of the circadian clock machinery, but in recent years, it has also emerged as an important part of the control of metabolism. It is well-known that there is a crosstalk between the circadian clock and metabolism, but the underlying molecular mechanism of this crosstalk is still elusive (Reinke and Asher, 2019). Haws et al. proposed a model in which circadian fluctuations need to be sensitive to metabolic flux, leaving another fraction of the epigenome unaffected. This will give the transcriptome metabolic flexibility, while critical chromatin functions, such as constitutive heterochromatin, remain unperturbed (Haws et al., 2020). Histone methylation could be one possible mechanism of crosstalk. SAM, FAD, and αKG seem to be influenced not only by circadian control but also by diet. In addition, HDM and HMT have a large range of kinetic parameters, thus responding differently depending on the metabolic flux and energy status (Mentch and Locasale, 2016). Furthermore, these enzymes seem to have circadian nuclear localization, a circadian peak of activity, and specific roles in metabolism. Thus, they could give feedback depending on the physiological need in a time-specific manner. However, further studies are needed to establish this relationship and determine whether these modifications and enzymes regulate specific metabolic or clock genes in a transcriptional feedback loop. Growing evidence also pinpoints the importance of nuclear metabolism in the epigenome; this includes the discovery of metabolic enzymes in the nucleus, the existence of SAM pools, and subcellular compartmentalization of metabolites (review in Boon et al. (2020)). These findings provide additional regulatory layers for the regulation of circadian rhythms and metabolism. Additionally, methylation patterns vary during aging. Recent studies demonstrate changes in DNA methylation on circadian clock genes and metabolic genes (Peng et al., 2019). Instead, histone methylation remains largely unexplored. Till now, only one study has demonstrated variations in histone methylation related to iron accumulation with age, which can affect metabolic patterns and the circadian clock. This appears to be directed by reduced AMP-modulated H3K9me3 in the *Per1* gene and H3K4me3 in the *Per2* promoter (Liu et al., 2022). Further investigation is needed to clarify how histone methylation intersects with the circadian clock to modulate the aging process and understand whether circadian transcription could be used, for example, as a predictor of healthy aging. Because circadian disruption is responsible for a widespread range of metabolic diseases, it is important to characterize the molecular mechanisms involved in the crosstalk between metabolism and circadian rhythms. These insights could provide novel strategies for the development of therapeutic targets for the treatment of highly prevalent diseases.
